# A scoping review of psychosocial functioning in young people with personality pathology: current evidence and methodological issues

**DOI:** 10.1186/s40479-026-00362-2

**Published:** 2026-07-30

**Authors:** Rasa Barkauskienė, Gabrielė Skabeikytė-Norkienė, Sophia Peter, Saskia Bamberger, Katja Bertsch, Chiara De Panfilis, Joost Hutsebaut, Andres Kaera, Mickey T. Kongerslev, Ueli Kramer, Mariana Mendoza-Alvarez, Emanuele Preti, Charlotte Rosenbach, Christian Schmahl, Michaela Swales, Svenja Taubner

**Affiliations:** 1 Institute of Psychology, Faculty of Philosophy, Vilnius University, Universiteto g. 9/1 LT-01513, Vilnius, Lithuania; 2 Institute for Psychosocial Prevention, Heidelberg University Hospital, Heidelberg, Germany; 3 Institute of Psychology, Heidelberg University, Heidelberg, Germany; 4 Lived Experience Group on the ESSPD Board, Ulm, Germany; 5 Department of Psychology, Julius-Maximilians-University Wuerzburg, Wuerzburg, Germany; 6 Unit of Neuroscience, Department of Medicine and Surgery, University of Parma, Parma, Italy; 7 Center of Research on Psychological disorders and Somatic diseases (CoRPS), Department of Medical and Clinical Psychology, Tilburg University, Tilburg, The Netherlands; 8 Medical Care Units, Mental Health and Substance Abuse Services, Wellbeing Services County of Kanta-Häme, Hämeenlinna, Finland; 9 Mental Health Services West, Region Zealand, Denmark; 10 Department of Psychiatry, University Institute of Psychotherapy, University of Lausanne, Lausanne, Switzerland; 11 Collaborative Antwerp Psychiatric Research Institute (CAPRI), University of Antwerp, Antwerp, Belgium; 12 Department of Psychology, University of Milano-Bicocca, Milan, Italy; 13 Faculty of Health, Heath and Medical University, Erfurt, Germany; 14 Department of Clinical Psychology and Psychotherapy, Freie Universität Berlin, Berlin, Germany; 15 Department of Psychosomatic Medicine and Psychotherapy, Central Institute of Mental Health, Medical Faculty Mannheim, Heidelberg University, Mannheim, Germany; 16 North Wales Clinical Psychology Programme Bangor University, Bangor, UK

**Keywords:** Personality disorder, Psychosocial functioning, Youth, Adolescence, Young people

## Abstract

**Background:**

Despite evidence that personality pathology often emerges during the transition from adolescence to adulthood and leads to lasting psychosocial impairment, research on how these impairments manifest and develop in young people remains limited.

**Objectives:**

This review aims to map existing literature on psychosocial functioning in individuals aged 12–25 with clinical or subclinical personality pathology, examining definitions and measures of functioning, its relationship with personality pathology, and the impact of early interventions, while identifying gaps for future research.

**Findings:**

A total of 37 studies were included: 14 cross-sectional, 7 longitudinal, and 16 on early interventions. In clinical samples, overall functioning was most assessed, whereas community studies focused on specific domains. Across studies, adolescents with personality pathology showed greater psychosocial impairment than both healthy controls and other psychopathology groups, both cross-sectionally and prospectively. Improvement in global psychosocial functioning has been documented across varying interventions but there was the lack of differences between treatment approaches.

**Conclusion:**

While emerging evidence highlights psychosocial dysfunction in young people with personality pathology, methodological limitations hinder comparisons across studies. Developmentally appropriate, culturally sensitive, and domain-specific assessments are needed to track psychosocial impairment alongside symptom progression in young people.

**Supplementary Information:**

The online version contains supplementary material available at https://doi.org/10.1186/s40479-026-00362-2.

## Introduction

The period of life between puberty and adult independence, referred to as the age of adolescence [1] or youth [2], is marked by significant changes in self-regulation abilities and interpersonal processes, both of which play a crucial role in mental health, overall functioning, and the formation of future developmental pathways. Furthermore, disturbances in self-regulation and relational processes involving self and others are viewed to constitute the core features of personality pathology [3], which typically has an onset during the period between puberty and emerging adulthood [4]. Across the lifespan, personality disorders (PD) appear to be a major contributor to disability, morbidity, premature mortality [5], and societal burden [6, 7]. Research indicates that their adverse effects on functioning commonly emerge before adulthood: early onset of borderline personality disorder (BPD) was linked to reduced educational and vocational outcomes as well as elevated healthcare costs already by the age of 20 [8]. The substantial health costs related to personality pathology stem not only from their severe symptomatology but also from marked impairments in psychosocial functioning [9, 10] as well as accumulation and continuity of symptoms and psychosocial impairment [11]. Furthermore, recovery of functioning has proved to be far more difficult to achieve than clinical remission [12]. While the link between PDs and psychosocial impairment has been extensively studied in adults [13], these parallels remain less understood during the early phase of the disorder trajectory. To the best of our knowledge, psychosocial impairment associated with BPD in adolescence has only been addressed in one systematic review [14]. Moreover, the conclusions of this review were based on four prospective studies only, which identified that adolescent BPD predicts short and long-term deficits in interpersonal, academic, occupational, and financial domains, even after accounting for psychiatric comorbidities or remission. Despite recent progress in research on the early stages of personality pathology, no additional reviews (systematic or otherwise) have been published on its associations with psychosocial functioning, to our knowledge.

Psychosocial functioning is a broad and multifaceted construct, making it challenging to define, delineate, and measure [15, 16]. It serves as an umbrella term encompassing various concepts and is generally understood as the extent to which individuals successfully interact with their environment across daily, occupational, and social domains [15]. Accordingly, specific areas of psychosocial functioning are measured, e.g., academic, work, interpersonal, recreation as well as social and occupational roles [17]. In the context of clinical settings, the American Psychiatric Association (APA) refers to the term ‘functional impairment’ as the degree to which symptoms interfere with an individual’s ability to adapt to the demands of multiple contexts, such as school, work, home, or interactions with other individuals, with assessments focusing on the global level of functioning. The World Health Organization (WHO) [18], in the International Classification of Functioning, Disability, and Health (ICF), defines functioning as the term “encompassing all body functions, activities, and participation” (p. 3) but also conceptualizes disability as an impairment occurring in any of the functional aspects - body (including physiological and psychological functions), activity (the ability to perform tasks), and participation (the extent to which an individual is involved in life). The version of ICF extended to children and youth (ICF-CY; [19]) adds environmental caveats to define a child’s level of functioning within the context of their environment. Despite definitional differences, both the APA and WHO advocate for a comprehensive assessment of functioning. Additionally, constructs, such as distress and quality of life are closely linked, as they often reflect the impact of symptoms [20], while other studies also include life satisfaction as well. To map the landscape of psychosocial functioning, Ro and Clark, [15] identified nearly 80 different measures capturing different aspects of functioning. However, this abundance of tools did not lead to a definite understanding or clear boundaries of psychosocial functioning [16].

With the increasing focus on early intervention for PD, there is a rising need for empirical understanding of which functional domains are most vulnerable in young people. Additionally, from the standpoint of emerging PD and early detection, it is crucial to consider individuals not only with clinical-level symptoms, but also with subclinical syndromes. To build a clearer and more comprehensive framework for this complex domain, it is essential to identify which specific functional constructs are being assessed and what methods are being used when examining psychosocial functioning in young people with varied levels of personality pathology. Previous efforts to identify a high-risk profile for PD in young people [21], as well as to explore factors contributing to its course during adolescence [22], have provided only a limited understanding of psychosocial impairments in young people. Additionally, assessing psychosocial functioning may clarify and measure the impact of early interventions on PD, making it a criterion not only for identifying dysfunction but also for tracking improvement. Therefore, accumulated empirical knowledge is needed to build a more comprehensive understanding of these dynamics.

A major recent development in the field of personality pathology is the conceptual and diagnostical change. Notably, both the DSM-5 Section III for Alternative Model for Personality Disorder (AMPD) [23] and ICD-11 [24] have adopted a dimensional approach. Within this framework, impairments in self and interpersonal functioning via the processes of identity, self-direction, empathy, and intimacy (Criterion A or level of personality functioning) are considered central to PDs, while maladaptive personality traits (Criterion B) are seen as stylistic manifestations of these core components. This shift has broadened the perspective on the relationship between personality pathology and psychosocial functioning. Since psychosocial functioning is embedded within PD’s diagnostic criteria in the categorical model (e.g., Section II in DSM-5), this raises the question of whether the two constructs are conceptually distinct or overlapping. The dimensional approach, however, offers a framework for viewing psychosocial disability as distinguishable from or a downstream consequence of impairments of personality processes across self and interpersonal domains [16, 25]. In adults, evidence suggests that the dimensional model-based assessments more consistently predict psychosocial outcomes than the number of categorically defined PD criteria [26–28]. In younger populations, the dimensional model of personality pathology, particularly Criterion A, is regarded as more sensitive for detecting early disruptions in personality development [29]. Consequently, it may more accurately predict emerging psychosocial impairments as personality pathology unfolds.

The current review aims to identify and document the scope of published literature examining psychosocial functioning among individuals aged 12 to 25 years or young people as, defined by the WHO^1^. In the developmental literature, this age range is commonly referred to as adolescence or youth [1, 2], encompasses transition from adolescence to adulthood and is marked by heightened vulnerability to mental-ill health [2]. The review focuses on young people who either meet diagnostic criteria for, or present symptoms of personality pathology as defined within both categorical and dimensional models of PD. Specifically, we seek to explore the relationship between personality pathology, as assessed through diagnostic categories, subclinical symptom counts, and dimensional models of PD (specifically Criterion A and Criterion B), and indicators of psychosocial functioning. Additionally, we examine the conceptual and methodological approaches used in the current body of research, evaluate the strength of the evidence, identify existing gaps, and offer recommendations for future studies. The objectives of this review, focusing on studies including participants aged 12 to 25 years, were the following:

1. To explore how psychosocial functioning is defined and operationalized in relation to PD or their features.
2. To identify what domains of (psychosocial) functioning impairments are examined and the measures used to assess them.
3. To map cross-sectional and longitudinal associations between clinical and subclinical presentations of personality pathology and indicators of psychosocial functioning.
4. To investigate how current early intervention efforts target psychosocial dysfunction, and what changes in psychosocial functioning (or its aspects) are reported as outcomes of these interventions.

## Method

To meet these objectives, a scoping review has been chosen as it represents an increasingly popular approach to summarizing the breadth and depth of a field. A scoping review is generally preferred over a systematic review when broader research questions are addressed, the literature has not been comprehensively reviewed yet, or when the topic is complex and heterogeneous in nature [30]. Scoping reviews also allow inclusion studies with diverse designs and methodologies, irrespective of quality, which is particularly valuable when evidence is limited evidence and broader topic coverage is needed [31]. The review was undertaken following the Preferred Reporting Items for Systematic Reviews and Meta-Analyses (PRISMA) guidelines, extended to scoping reviews (https://www.prisma-statement.org/scoping), and the updated methodological framework [32] was used for: (a) identifying the research question(s) and inclusion criteria, (b) searching and identifying relevant papers, (c) extracting and charting the data, (d) summarizing, and reporting the results. PRISMA guidelines application summary is provided in Table S1 (Supplementary Material). The review has not been preregistered.

### Information sources and search strategy

A literature search was carried out by the first and last authors after agreement on search terms. The search terms used in the PubMed and Scopus databases are listed in Table 1. A search through the reference lists of the selected papers was also made for additional resources. The search parameters were unlimited regarding the time of publication and allowed the period from inception to March 2026. We included studies from any geographical location, published in a peer-reviewed journal, and written in English. At the end of the search process, two researchers (GSN and SP) joined the first and last authors, forming two teams for extracting information.

**Table 1:** Search terms used in databases

PubMed
(“psychosocial functioning“[Title/Abstract] OR “psychosocial impairment“[Title/Abstract]) AND (“Adolescence“[Title/Abstract] OR “Adolescent“[Title/Abstract] OR “Youth“[Title/Abstract] OR “young people“[Title/Abstract]) AND (“personality disorder“[Title/Abstract] OR “personality pathology“[Title/Abstract] OR “Borderline“[Title/Abstract] OR “BPD“[Title/Abstract] OR “alternative model for personality disorder“[Title/Abstract]) AND (“Development“[Title/Abstract] OR “Intervention“[Title/Abstract] OR “Cross-sectional“[Title/Abstract] OR “longitudinal“[Title/Abstract] OR “cohort“[Title/Abstract] OR “prospective“[Title/Abstract] OR “retrospective“[Title/Abstract] OR “trial“[Title/Abstract] OR “clinical“[Title/Abstract] OR “community“[Title/Abstract])
Scopus
( TITLE-ABS-KEY ( psychosocial functioning ) OR TITLE-ABS-KEY ( psychosocial impairment ) AND TITLE-ABS-KEY ( adolescence ) OR TITLE-ABS-KEY ( adolescent ) OR TITLE-ABS-KEY ( youth ) OR TITLE-ABS-KEY ( young people ) AND TITLE-ABS-KEY ( personality disorder ) OR TITLE-ABS-KEY ( personality pathology ) OR TITLE-ABS-KEY ( borderline ) OR TITLE-ABS-KEY ( BPD ) OR TITLE-ABS-KEY ( alternative model for personality disorder ) AND TITLE-ABS-KEY ( development ) OR TITLE-ABS-KEY ( intervention ) OR TITLE-ABS-KEY ( cross-sectional ) OR TITLE-ABS-KEY ( longitudinal ) OR TITLE-ABS-KEY ( cohort ) OR TITLE-ABS-KEY ( prospective ) OR TITLE-ABS-KEY ( retrospective ) OR TITLE-ABS-KEY ( trial ) OR TITLE-ABS-KEY ( clinical ) OR TITLE-ABS-KEY ( community ))

### Eligibility criteria and study selection process

The eligibility criteria for the retrieved papers included the following characteristics related to (a) participants (research involving a population of young people aged 12–25 or mean age < 25); (b) concept ((i) focus on the indicators of personality pathology ranging from symptoms (features) to PD diagnosis and/or dimensional PD assessment) and (ii) psychosocial functioning or impairment); (c) content (examines the link of psychosocial functioning with personality pathology features or diagnosis (correlational, longitudinal links, group differences, or outcome of intervention).

Study selection was carried out by two pairs of authors (RB & GSN and SP & ST), who assessed the eligibility of the studies and extracted the information in the selected papers. The final selection of papers was made via the joint conference meeting of the reviewers. Any disagreements between the teams were resolved through discussion and joint review of the studies by all four researchers involved in the extraction process.

### Data collection process, data items, and synthesis

Data records were managed using Microsoft Excel. A standardized form was used to collect and extract information for the review. Extracted information included the following: study setting; study methodology; study population and participant demographic data; construct of personality pathology (features) and its assessment method; definition (construct) of psychosocial functioning and its measure; main results indicating (a) association (cross-sectional, longitudinal, or group differences) between personality pathology (features) and psychosocial functioning indicator(s); (b) change in psychosocial functioning (aspect) as a result of intervention. To summarize the findings, a narrative synthesis was conducted.

## Results

### Study characteristics

**Fig. 1:**
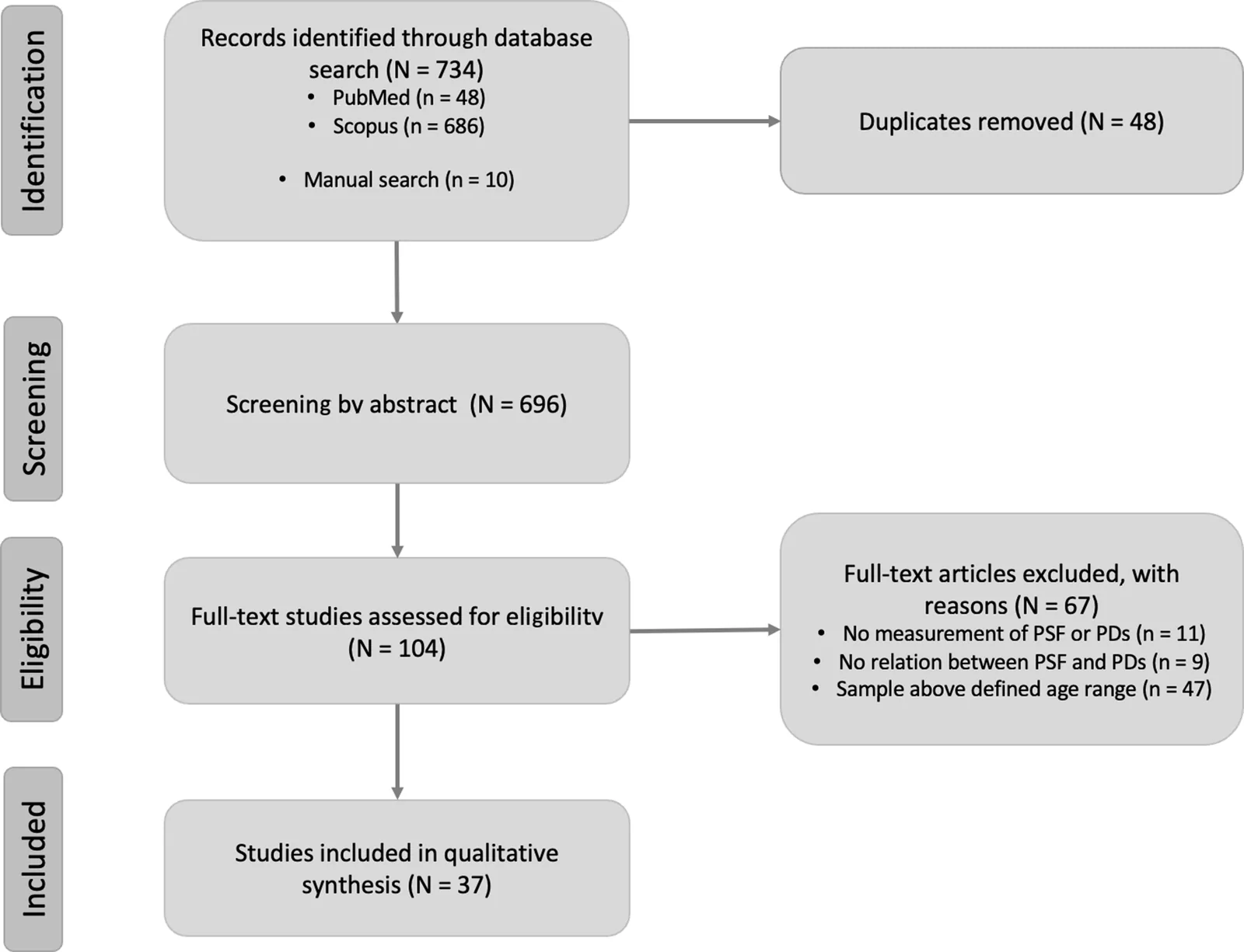
PRISMA diagram showing study selection process

The article selection process is depicted in Fig. 1. The initial search from PubMed and Scopus resulted in 734 articles, and 10 additional papers were added based on a manual search by reviewing the reference lists of the included papers. Following the removal of duplicates and the initial screening based on titles and abstracts, the inclusion criteria described above were applied to 104 full text articles. Among these, 67 articles were excluded based on the following criteria: (1) did not measure psychosocial functioning and/or personality pathology (features) (*n* = 11); (2) did not report any analysis linking personality pathology (features) to psychosocial functioning (*n* = 9); and (3) the sample did not include defined age range or mean age < 25 years (*n* = 47).

### General characteristics of the papers included

A total of 37 original studies met the selection criteria (see Table 2): 14 cross-sectional, 7 longitudinal, and 16 focused on early intervention. Most used clinical samples (*N* = 28; help-seeking individuals or participants diagnosed with PD recruited from inpatient or outpatient settings), while seven drew on community samples (participants recruited through schools or community settings), and two included both. Some publications reported overlapping participant samples.

**Table 2:** Characteristics of included studies

Author (year)	Country	Study design	Population	Sample characteristics	Personality pathology (feature) assessment approach and measure^a^	Psychosocial functioning domain measure^b^
Amminger et al. [33]	Australia	Intervention trial	Clinical, outpatient	*N* = 15; age range 13–25 ; M = 16.2, SD = 2.1); 93.3% female	Categorical approach; BPD assessment based on DSM-IV criteria	GAF
Barkauskienė et al. [34]	Lithuania	Cross-sectional	Community	*N* = 379; age range 11–18 (M = 14.69, SD = 1.74); 55.9% female	Categorical approach; Self-report assessment of borderline personality symptoms; BPQ	YSR 11–18 (Social Problems Scale); number of close friends; achievement (question about grades received) PAMS; SWLS
Barkauskienė et al. [35]	Lithuania	Cross-sectional	Clinical and community	*N* = 1048; age range 12–18 (M = 14.56; SD = 1.59); community-based adolescents (*N* = 855; M = 14.44, SD = 1.60), 62.5% female and clinical group of 193 adolescents (M = 15.11, SD = 1.41; 79.8% female	Categorical and dimensional approaches; Self-report assessment of BP features, Criterion A and Criterion B; BPFSC-11, LoPF-Q 12–18, PID-5-BF	YSR 11–88 (Social Problems Scale)
Barkauskienė et al. [36]	Lithuania	Longitudinal	Community	*N* = 855; age range 11–18 (M = 14.44, SD = 1.60); 62.5% female	Dimensional approach; Self-report assessment of Criterion A and Criterion B; LoPF-Q 12–18, PID-5-BF	YSR 11–18 (Social Problems Scale); MPVS-r24 (Social Rebuff Scale); PAMS; SWLS
Baumer et al. [37]	Switzerland, Germany	Intervention study	Clinical, outpatient	*N* = 44; age range 13–19 (M = 16, SD = 1.4); 97.7% female	Categorical approach; BPD assessment based on SCID-II	C-GAS
Beck et al., [38]	Denmark	Intervention study	Clinical	*N* = 111; age range 14–17 (M = 15.7, SD = 1.1); 99% female	Categorical approach; BPD assessment based on CI-BPD	C-GAS
Beeney et al. [39]	United States	Longitudinal, annual assesments	Community	*N* = 2450; age range 8–15; 100% female	Categorical approach; BPD assessment based on IPDE-Screen	C-GAS; school functioning (two questions)
Biskin et al. [40]	Canada	Longitudinal, 2 assessments	Community	*N* = 906; age range 11–13 (M = 12.34, SD = 0.48); 55% female	Categorical approach; Self-report assessment of BP features; BPQ	CIS-Y
Calvo et al. [41]	Spain	Cross-sectional	Clinical, outpatients	*N* = 216; age (M = 24.02, SD = 9.07); 91.9% female; *N* = 69 (a subsample of adolescents with BPD, age range 14–18).	Categorical approach; BPD assessment based on SCID-II; Self-report assessment based on BPFSC-11	FAST
Cavelti et al. [42]	Switzerland, Germany	Intervention study, 3 assessments	Clinical, outpatient	*N* = 127; age (M = 15.20, SD = 1.60); 94.5% female	Categorical approach; BPD assessment based on SCID-II	Composite score of the Level of Functioning based on SOFAS and GAF
Cavelti et al. [43]	Switzerland, Germany	Intervention study, 3 assessments	Clinical, outpatient and inpatient	*N* = 63 in combined inpatient/outpatient group; Mage = 15.17, SDage = 1.41; 87% female. *N* = 115 in outpatient only group, Mage = 14.73, SDage = 1.49; 94% female	Categorical approach; BPD assessment based on SCID-II	GAF
Cavelti et al. [44]	Switzerland, Germany	Cross-sectional	Clinical	*N* = 706, age range 11–18 (M = 15.4, SD = 1.54); 80.88% female	Dimensional approach; Criterion A assessment based on STiP-5.1	SOFAS; GAF
Chanen et al. [45]	Australia	Cross-sectional	Clinical, outpatient	*N* = 177; age range 15–18 (M = 16.24, SD = 0.94); 68.4% female	Categorical approach; BPD assessment based on SCID-II	SOFAS; HoNOSCA (items on peer and family relationships, self-care, school attendance); questions about the number of days out of role
Chanen et al. [46]	Australia	Intervention trial	Clinical, outpatient	*N* = 86; age range 15–18 (M = 16.4; SD = 0.9); 76% female	Categorical approach; BPD assessment based on SCID-II	SOFAS
Chanen et al. [47]	Australia	Intervention trial	Clinical, outpatient	*N* = 139; age range 15–25 (M = 19.1, SD = 2.8); 81.3% female	Categorical approach; BPD and other PD assessment based on SCID-II	SOFAS; NEET status; IIP-CV; SAS-SR
Chanen et al. [48]	Australia	Cross-sectional	Clinical, outpatient	*N* = 141; age range 15–25 (M = 19.3, SD = 2.9); 64.5% female	Categorical approach; BPD assessment based on SCID-II	SOFAS
Ghinea et al. [49]	Germany	Cross-sectional	Clinical, outpatient	*N* = 368; age range 12–17 (M = 14.92, SD = 1.43); 91.03% female	Categorical approach; BPD assessment based on SCID-II	GAF
Gleeson et al. [50]	Australia	Intervention study	Clinical, outpatient	*N* = 16; Age range 15–25 (M = 18.4; SD = 2.9); 82.2% female	Categorical approach; BPD and antisocial PD assessment based on SCID-II	SOFAS
Gleeson et al. [51]	Australia	Intervention study, 3 months follow-up	Clinical, outpatient	*N* = 8; age range 16–25 (M = 18.9; SD = 3.2); 87.5% female	Categorical approach; BPD severity	SDS
Hessels et al. [52]	Netherlands	Cross-sectional	Clinical	*N* = 246; age range 12–25 (M = 19.22, SD = 2.76); 81.7% female	Categorical and dimensional approaches; Self-report assessment of BPD features, Criterion A and Criterion B; SCID-II-PQ; BPD	DML
Jorgensen et al. [53]	Denmark	Intervention study	Clinical	*N* = 111; age range 14–17 (M = 15.7, SD = 1.1); 99% female	Categorical approach; BPD assessment based on SCID-5-PD	C-GAS
Jorgensen et al. [54]	Denmark	Intervention study, 5-year follow-up	Clinical	*N* = 97; age range 19–24 (M = 21.5, SD = 1.2); 99% female	Categorical approach; BPD assessment based on SCID-5-PD	WSAS, NEET status
Kaess et al. (2012)	Germany	Cross-sectional	Clinical	*N* = 87; age range 13–18; (M = 16.23, SD = 1.54 in BPD group; M = 15.45, SD = 1.35 in non-BPD group); 100% female	Categorical approach; BPD assessment based on SCID-II	C-GAS
Kramer et al. [55]	United States	Cross-sectional	Clinical and community (as control group)	*N* = 164; age range 13–17 (M = 15.7, SD = 1.2; 94.2% female in BPD group; M = 14.8, SD = 1.4; 83.3% female in control group)	Categorical approach; BPD assessment based on KID-SCID, DIB-R, & CI-BPD	BIS-AV
Mehlum et al. [56]	Norway	Randomized trial	Clinical	*N* = 77; age range 12–18 (M = 15.6, SD = 1.5); 88.3% female	Categorical approach; BPD assessment based on SCID-II	C-GAS
Mehlum et al. [57]	Norway	Randomized trial, 1-year follow-up	Clinical	*N* = 75; 97% participation rate for the 1-year follow-up rate of the original sample [56]	Categorical approach; BPD assessment based on SCID-II	C-GAS
Mehlum et al. [58]	Norway	Randomized trial, 3-year follow-up	Clinical	*N* = 71; 92% participation rate for the 1-year follow-up rate of the original sample [56]	Categorical approach; BPD assessment based on SCID-II	C-GAS
Palermo et al. [59]	Switzerland	Longitudinal follow-up	Clinical, outpatient	*N* = 227; age range 12–17 (M = 15.25; SD = 1.48); 85.02% female	Dimensional approach; Criterion A assessment based on STiP-5.1	SOFAS
Schmeck et al. [60]	Switzerland, Germany	Intervention study	Clinical	*N* = 60; age range 13–19 (M = 15.78, SD = 1.35); 93% female	Categorical approach; BPD assessment based on SCID-II	C-GAS; CIS-P; CIS-P
Sharp et al. [25]	Lithuania	Cross-sectional	Community, clinical	Community: *N* = 379; age range 11–18 (M = 14.70; SD = 1.74); 55.9% female Clinical: *N* = 74; age range 11–17 (M = 15.05; SD = 1.47); 71.6% female	Categorical and dimensional approaches; Identity as part of Criterion A assessment based on AIDA; BPD assessment based on self-report BPQ	SDQ (Peer Problems Scale); PAMS; SWLS
Thompson et al. [61]	Australia	Cross-sectional	Clinical, outpatient	*N* = 266; age range 15–25. BPD features group *N* = 111; Mage = 18; 56.66% female; 1–4 BPD features group *N* = 155; Mage = 17.72; 65.16% female	Categorical approach; BPD assessment based on SCID-II	SOFAS
Thompson et al. [62]	Australia	Cross-sectional	Clinical, outpatient	*N* = 499; age range 15–25 (M = 17.98, SD = 2.67); 70.4% female	Categorical approach; BPD assessment based on SCID-II	SOFAS
Wertz et al. [63]	Great Britain	Longitudinal	Community	*N* = 2232; age range 12–18 at baseline; 51% female.	Categorical approach; interview-based assessment of BPD symptoms; composite of items from SWAP-200-A and ASEBA	NEET status; GCSE; MSPSS; SWLS; Official crime records
Winograd et al. [64]	United States	Longitudinal	Community	*N* = 748; age range at baseline 9–18 (M = 13.74, SD = 2.56); 50% female	Categorical approach; Self-report assessment of borderline symptoms; CIC-SR	Questions created by study authors on role function; LWSMAT; one item on life satisfaction adapted from social indicators well-being measures
Wright et al. [65]	United States	Longitudinal	Community	*N* = 2450; age range 14–17; 100% female	Categorical approach; Self-report assessment of BPD symptoms; IPDE-BOR	C-GAS; parent ratings of achievement level; self-reports of activity involvement; SSRS; ASAI; POPS
Wyssen et al. [66]	Switzerland, Germany	Cross-sectional	Clinical	*N* = 526; age range for this analysis: 14–17 (M = 15.41, SD = 1.52); 78.9% female	Categorical and dimensional approaches; Criterion A assessment based on STiP-5.1; BPD assessment based on SCID-II	C-GAS; SOFAS
Zanarini et al. [67]	United States	Randomized trial	Clinical	*N* = 50; age range 18–30 (M = 19.3, SD = 1.4); 100% female	Categorical approach; BPD assessment based on DIPD-IV and DIB-R	SDS

Note:

a. AIDA = Assessment of Identity Development in Adolescence; BPFSC-11 = Borderline Personality Features Scale for Children; BPQ = Borderline Personality Questionnaire; BSL = Borderline Symptom List; CI-BPD = Childhood Interview for DSM-IV Borderline Personality Disorder; CIC-SR = The Children in the Community Self-Report; DIB-R = The Revised Diagnostic Interview for Borderlines; DIPD-IV = The Diagnostic Interview for DSM-IV Personality Disorders; IPDE-BOR = The International Personality Disorders Examination; IPDE-Screen = The International Personality Disorders Examination-screen version; KID-SCID = The Structured Clinical Interview for DSM-IV Childhood Diagnoses; LoPF-Q 12–18 = Levels of Personality Functioning Questionnaire 12–18; LPFS = The Level of Personality Functioning Scale 2.0; SCID-II = Structured Clinical Interview for DSM-IV Axis II Disorders; SCID-II-PQ BPD = SCID-II Questionnaire for BPD; PID-5-BF = Personality Inventory for DSM-5 Brief Form; SCID-5-PD = The Structured Clinical Interview for DSM-5 Personality Disorders; STiP-5.1 = Semi-Structured Interview for Personality Functioning DSM-5; SWAP-200-A = Shedler-Westen Assessment Procedure 200-item Q-Sort for Adolescents

b. ASAI = Adolescent Sexual Activity Index; BIS-AV = Background Information Schedule: adolescent version; C-GAS = Children’s Global Assessment Scale; CIS-P = Columbia Impairment Scale Parent version; CIS-Y = Columbia Impairment Scale Youth version; DML = Developmental Milestones List; GCSE = General Certificate of Secondary Education; FAST = Functioning Assessment Short Test; HoNOSCA = Health of the Nation Outcome Scales for Children and Adolescents; IIP-CV = Inventory of Interpersonal Problems Circumplex Version;; LWSMAT = Locke-Wallace Short Marital-Adjustment Test; MPVS-r24 = Multidimensional Peer-Victimization Scale Revised-24; MSPSS = Multidimensional Scale of Perceived Social Support; PAMS = Perceived Academic Motivation Scale; POPS = Perception of Peers and Self inventory; SAS-SR = Social Adjustment Scale Self-Report; SWLS = Satisfaction with life scale; SDQ = Strengths and Difficulties Questionnaire; SOFAS = Social and Occupational Functioning Assessment Scale; SDS = Sheenan Disability Scale; ; SSRS = Social Skills Rating Scale; WSAS = Work and Social Adjustment Scale; YSR 11–18 = Youth Self-Report

Table 2 summarizes sample characteristics, detailing author, publication year, study location, research design, participant characteristics, personality pathology approach and measures of psychosocial functioning measures. Mean participant age ranged from 13.7 to 19.3; age ranges were used when means were unavailable. Across studies, the proportion of female participants ranged from 56.7 to 100% in clinical samples and 50–100% in community samples. Sample sizes varied from 8 to 2,450.

The 37 studies originated from 12 countries. Most studies were conducted in Australia (*n* = 9) and Germany (*n* = 8), followed by Switzerland (*n* = 7), the United States (*n* = 5), and Lithuania (*n* = 4). There were also studies from Denmark (*n* = 3), Norway (*n* = 3), and one study each from the United Kingdom, Canada, Spain, and the Netherlands.

Personality pathology assessment methods included clinical interviews for PDs based on both categorical and dimensional models (see Table 2). One study included official medical records. Additionally, self-report measures for borderline features (symptoms) and the level of personality functioning were used. Despite this variation, most studies (30/37) used categorical approach; four combined categorical and dimensional approaches, and only three used only dimensional approach.

### Psychosocial functioning definitions and measurement

Regarding the construct of psychosocial functioning, only five studies referred to a clear definition, with three of them using Skodol’s [13] definition as “a person’s ability to carry out roles and perform activities in daily life, including in social or interpersonal, school or work, recreational or leisure, and basic (i.e., self-care, communication, mobility) functional realms” (p. 33). Other studies referred to good psychosocial functioning and underscored the complexity and multifaceted nature of the construct.

Psychosocial functioning was assessed using both self-report and clinician-rated measures (Table 2). In clinical populations, most tools assessed overall functioning, with the Global Assessment of Functioning Scale (GAF; APA, 1994) or the Children’s Global Assessment Scale [68] being the most frequently used (*n* = 16) as well as the Social and Occupational Functioning Assessment Scale [69] (*n* = 11), which targets social and occupational domains to combine in a single global functioning score, but also others, e.g., the Background Information Schedule: adolescent version [70], Sheehan Disability Scale (SDS; Sheehan, Harnett-Sheehan & Rai, 1996). Only six studies in the clinical samples included domain-specific measures of functioning. Furthermore, one study used a novel self-report tool to evaluate the attainment of developmental milestones, thereby assessing overall age-adequate psychosocial functioning across personal, social, and societal domains [71], and recently, the Functioning Assessment Test [72] has been validated among young people with personality disorder in Spain [41].

Community studies more often used multi-domain assessments of functioning. Only three incorporated a measure of overall (global) functioning, using the C-GAS and Columbia Impairment Scale [73]. Most community-based assessments targeted social or interpersonal functioning (*n* = 8) and academic or school functioning (*n* = 7). Other domains, such as occupational (i.e., roles, participation, NEET status – Not in Education, Employment, or Training), societal functioning (e.g., crime records), and subjective assessments (i.e., life satisfaction) were also addressed in individual studies (see Table 2).

### Cross-sectional studies

#### Clinical samples

 Key findings are summarized in Table S2 (Supplementary Material). Twelve publications reported cross-sectional analyses in clinical samples. Four studies [45, 48, 55, 74] examined global functioning in young people with clinically significant personality pathology and consistently found that participants with BPD diagnosis showed greater impairments in global functioning – ranging from moderate to severe – than both clinical comparison groups or healthy controls. Subthreshold BPD manifestations were likewise associated with moderate impairment in overall functioning [49, 61].

Three studies examined domain-specific psychosocial functioning and reported greater interpersonal impairment among adolescents with personality pathology compared with clinical or healthy control groups [41, 45, 55]. Regarding other domains, Kramer et al. [55] found large differences in occupational functioning between adolescents with BPD and healthy controls. In contrast, Chanen et al. [45] observed no differences in school attendance among adolescents with PDs and those with other mental conditions, although patients with BPD had more days out of role due of truancy.

Two studies compared categorical and dimensional models in relation to overall functioning. Wyssen et al. [66] found that adolescents exceeding diagnostic threshold on Criterion A, BPD, or both showed higher psychosocial impairment than the non-PD group, with the most severe impairment observed among those elevated on. Hessels et al. [52] further showed that Criterion A and Criterion B explained more variance in age-adequate functioning than BPD symptoms. Overall, studies reported moderate associations between global functioning and personality pathology across both categorical and dimensional models [41, 44, 49, 52, 62, 66].

Psychosocial impairment appeared uniquely linked to personality pathology. Chanen et al. [45] found that BPD with comorbid PDs predicted poorer global functioning and peer relationships beyond psychiatric diagnoses, while daily functioning was explained solely by BPD after controlling for Axis I disorders and other PDs. Thompson et al. [62] reported that BPD explained 11.7% of the variance in global functioning, and Barkauskienė et al. [35] reported that Criterion A accounted 7.8% of the variance in social functioning. Ghinea et al. [49] showed that BPD severity and diagnosis explained the association between childhood maltreatment with the level of global functioning in adolescents with NSSI at the same variance as depression.

#### Community samples

In adolescents from community samples, BPD symptom severity was linearly associated with psychosocial impairment, with greater symptom severity linked to poorer functioning [34]. Adolescents in the highest severity group (19.7%) reported the greatest social difficulties, fewer close friendships, lower academic motivation and achievement, and the lowest life satisfaction, whereas the moderate group showed inconsistent differences from other groups in friendships or achievement.

Across studies, the strongest associations were observed for social functioning [35] and life satisfaction [25], while associations with academic functioning and number of close friends were weaker [34]. BPD features explained statistically significant though small additional variance in social problems (1.1%), academic achievement (1.2%), and life satisfaction (2.8%) [34], while Criterion A accounted for 7.8% of variance in social problems [35].

### Longitudinal studies

#### Clinical samples

Only one study [59] examined whether global psychosocial functioning predicted changes in personality functioning and BPD diagnosis over two years. Baseline psychosocial functioning did not predict diagnostic remission or overall changes in personality functioning, but higher baseline functioning was associated with less improvement in self-direction.

#### Community samples

Six studies examined longitudinal associations between personality pathology and psychosocial functioning. Most studies focused on personality pathology (features of) as predictors of functioning over time [36, 40, 63, 64, 65], while also describing functioning trajectories [64], indicating bidirectional links [65], and examining psychosocial functioning as predictor of later BPD symptoms [39].

Early BPD symptoms predicted pooter outcomes at 10-year follow up, including lower role function, reduced social support, and lower life satisfaction (all regression coefficients corresponded to small effect sizes) [64]. They also uniquely predicted low educational qualifications (IRR = 1.18), risky sexual behavior (IRR = 1.17), and official crime records (IRR = 1.19), controlling for sex and initial levels of psychopathology at age 12 [63]. In Wright et al. [65] study, individual differences in initial BPD levels uniformly correlated with all functioning domains (Mdn *r* = .50; range = 0.21-0.61) across a three-year period from 14 to 17 years old girls. Higher psychosocial impairment was additionally linked to persisted self-harm alongside elevated borderline symptoms from ages 11–13 to 18–20 [40].

Winograd et al. [64] further showed that individuals with a diagnostic level of early BPD features (2 SD above the mean) had persistently poorer role functioning, declining life satisfaction, and diminished social support between ages 15 and 25, although the quality of their closest relationships remained stable. Prior role functioning explained approximately 30% of the association between role functioning and BPD symptoms through age of 25. Wright et al. [65] observed declining academic functioning was observed alongside increasing global functioning impairment and sexual activity. Within this sample, elevations in BPD symptoms were accompanied by worsening psychosocial functioning, particularly in interpersonal and sexual domains, and vice versa, supporting bidirectional links. Using the same data set, [39] found that global functioning (along with other variables) at ages 14–15 predicted BPD symptoms at ages 16–18, whereas functioning from earlier age periods (at 8–10 and 11–13 years old) was not predictive of BPD symptoms at ages 14–15.

The only study used dimensional approach and showed that longitudinal associations between baseline Criteria A and B and multi-domain psychosocial functioning remained statistically significant one year later and were comparable in strength to baseline cross-sectional associations [36]. Criterion A uniquely predicted later social difficulties, social rebuff, and life satisfaction, whereas Criterion B uniquely explained social and psychoticism domain added to social rebuff.

### Change in psychosocial functioning as an outcome of interventions

Sixteen studies evaluated a variety of (early) intervention programs or approaches. Of these, fourteen assessed overall psychosocial functioning as an outcome, while one focused on changes in domain-specific functioning. Most studies reported improvements in functioning following treatment, although some negative findings were noted [67].

Three studies [38, 54, 75] from different phases of the Mentalization-based treatment in groups (MBT-G) randomized trial for adolescents meeting at least four DSM-5 BPD criteria found improvement in global functioning by treatment completion, with participants moving from major to moderate impairment [38], However, no further gains occurred at the 3-month, 12-month follow-ups, or 5-year follow-ups, and functioning remained at a moderate level of functional impairment [53, 54]. Furthermore, MBT-G did not show superior effects on global functioning compared with treatment as usual. A similar pattern was observed for Dialectical Behavior Therapy for Adolescents (DBT-A) in adolescents with self-harming behavior and borderline symptoms: functioning improved within moderate impairment range achieved at the end of treatment and remained stable at the 1- and 3-year follow-ups [56–58].

Baumer et al. [37] and Schmeck et al. [60] compared Adolescents Identity Treatment [76] vs. DBT-A and found substantial and comparable improvements in global functioning across both treatment conditions, with similarly large effect sizes. Higher baseline positive treatment outcome expectations were linked with greater gains in psychosocial functioning [37].

Two studies [42, 43] reported continued improvements in global functioning among adolescents who received (1) Cutting Down program [77] only; (2) those who continued with DBT-A following CDP; and those (3) who were offered but declined DBT-A after CDP. No clinically significant differences in psychosocial functioning at follow-up were found between those who accepted versus declined DBT-A [42]. Greater improvements at 1- and 2-year follow-ups were observed in outpatients than in a mixed inpatient-outpatient sample [43].

An early-intervention trial by Chanen et al. [46] compared Cognitive Analytic Therapy (CAT) as compared with standardized good clinical care (GCC). Both groups improved in global functioning, with no noticeable differences at 2-year follow-up, despite the expected rate of improvement being moderately faster for GCC group. In a later trial, Chanen et al. [47] examined social and interpersonal functioning across three treatment conditions: (1) Helping Young People Early (HYPE) care model combined with CAT, (2) HYPE combined with befriending and (3) Youth Mental Health Services (YMHS) service model combined with befriending. Social functioning improved significantly over the 12-month period (from 19.3% to 23.8%), but neither the service model (HYPE vs. YMHS) nor intervention type (CAT vs. befriending) demonstrated superiority.

Two studies focused on patients with both BPD and first-episode psychosis (FEP). Gleeson et al. [50] reported improved global functioning following HYPE combined with specialist FEP treatment (SFET) compared with SFET alone, at treatment completion and 6-month follow-up in the. Amminger et al. [33] investigated patients receiving either 0.5 g of concentrated marine fish oil showed significant improvement in global psychosocial functioning, increasing from 55.7 to 75.6 (Cohen’s d = 1.59) compared with placebo.

Finally, Gleeson et al. [51] reported improved global functioning scores among young people with BPD whose caregivers and friends participated the Kindred online intervention program. Although changes were not statistically significant, the effect size was large.

## Discussion

This scoping review examined empirical studies on psychosocial functioning among young people aged 12 to 25 in relation to personality pathology, as defined by both categorical and dimensional PD models. Thirty-seven publications were identified, indicating a growing scholarly interest, particularly in recent years, with 23 studies published since 2020. In general, findings suggest that young individuals with subclinical or clinical levels of symptoms of personality pathology experience significant difficulties across multiple areas of psychosocial functioning. Although studies vary in design and methodology and the overall evidence base remains limited, the main findings and issues stemming from this review merit attention.

First, in clinical samples, global psychosocial functioning has been most extensively examined and has consistently been found to be impaired, ranging from severe to moderate [38, 45, 48, 60, 74]. However, these levels did not differ between those with BPD and those with other PDs [45] or first-episode psychosis [48]. The number of BPD criteria was found to explain the (unique) variance in functioning [62], mirroring findings in adult samples [17, 78]. At the same time, these results may underscore potential limitations associated with global functioning measures, particularly GAF and C-GAS, that highly tend to conflate symptom severity with functional impairment. Consequently, observed associations may partly reflect measurement overlap, rather than true predictive relationships. Nevertheless, studies using the SOFAS, which assesses functioning independently of psychopathology, have reported comparable levels of impairment, lending further support for the robustness of these findings.

Global functioning was the most frequently assessed outcome of intervention. Although findings were mixed, most studies indicated potential post-treatment improvement [42, 46, 50]. Some evidence suggested greater gains among outpatients than inpatients [43], while others reported recovery in a subset of participants [60]. Improvements at treatment completion generally remained stable long term [54, 57, 58, 75], extending into adulthood and beyond age 25 [79]. However, given the wide variation of interventions, ranging from nutritional supplementation to psychosocial care, it remains unclear whether gains were attributable to the interventions themselves, natural developmental maturation [75], other mechanisms [80], or limitations in outcome measurement. The lack of differences between treatment approaches suggests the need for more precise functional assessments. Inclusion of domain-specific functioning measures may offer potential value for understanding the mechanisms underlying change across diverse interventions, yet only two studies used these so far [47, 67].

Second and relatedly, studies identified in our scoping review highlight an asymmetry in the measures used across clinical and community samples. Clinical research has primarily relied on global psychosocial functioning assessments, whereas community-based studies have focused more on domain-specific functioning, particularly social (interpersonal) functioning. Although both contexts consistently link BPD features and overall social difficulties, findings on specific aspects of social functioning and personality pathology are mixed [34, 64, 65], with some studies reporting no significant differences in peer relationship quality in adolescents with BPD compared to healthy controls [55]. Overall, the literature in this essential domain remains fragmented, with most studies focusing on discrete components, such as social isolation [63], social skills, and social support [64, 65], rather than examining these aspects in an integrated manner. This highlights the need for research that captures the complex, multifaceted nature of social and interpersonal functioning in young people with personality pathology. A related scoping review [81] similarly highlights the need for research capturing the full range of relevant interpersonal and social constructs.

Next, academic or educational functioning is a key domain for young people, yet only 10 out of the 37 reviewed studies addressed it. Though more often addressed in community samples, just two studies examined it clinical samples [45, 55]. Academic underachievement is a major contributor to NEET status [11] and explains approximately 30% of the association between role functioning (defined as being a student, working, or neither) and BPD features over 10 years [64]. Given its broad and cumulative impact [11, 64], academic functioning among young people across the spectrum of personality dysfunction remains under-researched, with the existing evidence being fragmented, precluding robust conclusions. Some education- and school-related constructs were classified under the vocational and/or occupational functioning instead [54, 55, 64]. As individuals in this age group are often studying and working simultaneously, there is limited agreement on where to draw the line between academic, school-related, and occupational/vocational functioning.

These findings highlight several methodological issues stemming from this review. First, the predominant reliance of global psychosocial functioning measures across many studies rather than targeting specific domains of functioning is noted. This imbalance is particularly pronounced when comparing clinical and community samples. Although a difference that can be attributed to diagnostic assessment methods, it may underscore a broader challenge in defining and measuring psychosocial functioning in the context of personality dysfunctions highlighted by other reviews in the field [17]. While tools such as GAF, C-GAS, and SOFAS offer information on overall psychosocial functioning, they mask the identification of where the functioning gaps may lie and limit understanding of the multidimensional nature of psychosocial functioning. Our review supports this concern, revealing that functioning trajectories are heterogeneous across domains, rather than uniform or linear [64, 65]. Domain-specific assessments are particularly valuable in early stages of personality pathology, as they may detect the progression of dysfunction alongside symptoms development in line with the clinical staging model [82]. As mentioned above, they are also essential for understanding change processes and how different areas of functioning interact within socioecological network [83]. This requires robust domain-specific assessments across different age groups and cultural contexts.

Second, the diversity of assessment measures limits comparability across studies, highlighting the need for standardized methods applicable to both subclinical and clinical presentations. As noted earlier, the ICF-CY conceptual framework can provide a common language for describing functioning, while the WHO Disability Assessment Schedule 2.0 (WHODAS 2.0; [84]), recommended by the American Psychiatric Association in the DSM-5 [85], may serve as a standardized assessment tool. WHODAS 2.0 evaluates overall impairment across six key domains: understanding and communicating, mobility, self-care, getting along with others, life activities (e.g., household, work, and/or school responsibilities), and participation in society [84] thereby aligning with the ICF-CY model of body functions, activities, and participation. Its broader adoption could facilitate more consistent implementation of the ICF framework across studies. Furthermore, WHODAS 2.0 has been endorsed by an expert consensus group as a recommended outcome measure in personality to improve consistency across studies [86]. Beyond the ICF framework, some authors have argued that quality of life represents a complementary construct that captures the subjective impact of symptoms on functioning [20]. In particular, health-related quality of life has been proposed as an indicator of recovery process [86]. Given that addressing psychosocial dysfunction in PD inherently involves a recovery-oriented perspective [78], future studies should incorporate (health-related) quality of life alongside the broader dimensions of psychosocial functioning outlined above.

Thirdly, the review highlighted a lack of longitudinal studies, particularly in clinical samples; only one study employed a longitudinal design and it was conducted within the context of intervention [59]. Longitudinal research covering the full spectrum of personality pathology in both clinical and community populations is needed to clarify the developmental and potentially bidirectional associations between personality dysfunction and psychosocial functioning. Using domain-specific, multidimensional assessments of psychosocial functioning may help identify the mechanisms underlying these associations and track how these associations unfold over time. Emerging evidence that psychosocial functioning may also predict personality dysfunction [39, 59] broadens the focus of intervention by suggesting that functioning should be targeted not only as an outcome of symptoms reduction but also as a potential mechanism of change. Contextualizing psychosocial functioning as both an outcome, predictor, and/or a dynamic process variable may further enhance understanding the trajectories of change and recovery. This perspective is also consistent with developmental psychopathology frameworks, which emphasize the ongoing, reciprocal interactions between individual characteristics and developmental contexts.

Fourth, as dimensional models of personality disorder become central to both the DSM-5 and ICD-11, research examining how dimensional personality pathology relates to psychosocial functioning remains limited. Emerging evidence, including the studies reviewed here and findings from adults (e.g., [26]), suggests meaningful associations, highlighting the need for further research in this area.

### Limitations

Several methodological features of the search strategy and review approach may have affected the breadth and completeness of the evidence identified and should be considered when interpreting the findings. Given the broad scope of the review, methodological decisions were made to balance comprehensiveness with feasibility and ensure a manageable and reproducible screening process. First, it primarily relied on the explicit term *psychosocial functioning*, although this construct is often indexed under a broader range of related terms across databases; consequently, some relevant studies may not have been captured. Similarly, personality pathology was largely identified using the umbrella term *personality disorder*, without systematically including specific PD subtypes, except for BPD. In addition, the absence of standard truncation and controlled vocabulary terms, together with restrictions to Title/Abstract fields, English-language publications, and peer-reviewed sources, may have further reduced search sensitivity. Additionally, as this was a scoping review, no methodological quality appraisal of included studies was conducted; therefore, no conclusions can be drawn regarding the consistency or robustness of the findings.

Finally, the evidence is derived almost exclusively from high-income Western countries with relatively similar healthcare systems and cultural contexts. Consequently, the findings may not fully generalize to populations in other cultural, socioeconomic, or healthcare settings, where the presentation of personality pathology, psychosocial functioning, and access to services may differ.

## Conclusions

Current research offers limited evidence on psychosocial functioning in young people with personality pathology, spanning from subclinical traits to clinically diagnosed disorders. This limitation is largely driven by the use of heterogeneous measurement tools across studies that include both clinical and community samples, which reduces comparability and hinders synthesis of findings. Available evidence suggests that psychosocial impairments associated with personality disorders emerge early in their development; however, specific indicators that reliably predict vulnerability to psychosocial impairment, particularly across distinct functional domains, remain insufficiently investigated.

There is a significant gap in how psychosocial functioning in young people personality pathology is conceptualized and assessed. Greater adoption of the ICF-CY as a common conceptual framework for defining and assessing disability and functioning, and as a guiding structure for organizing and synthesizing the evidence in future reviews, would promote greater consistency in terminology, measurement, and interpretation across studies. More consistent application of functioning assessment tools informed by the ICF-CY and recommended by DSM-5, alongside the standardized outcome set s recommended for PDs [86] may further enhance the ability to capture the multidimensional nature of psychosocial functioning more effectively.

At present, intervention programs targeting personality pathology in young people rarely prioritize psychosocial outcomes, such as preventing school dropout or improving social participation. Furthermore, our review also identified no studies that incorporate environmental modifications to support participation among young people with personality dysfunction, although a promising intervention trial has recently been initiated [87]. This gap is especially concerning during adolescence, a critical developmental window in which psychosocial functioning is central to healthy development. Failure to adequately address psychosocial functioning at this stage may undermine efforts to prevent long-term disability.

## Supplementary Information

Below is the link to the electronic supplementary material.

Supplementary Material 1

Supplementary Material 2

## Data Availability

No datasets were generated or analysed during the current study.

## Footnotes

1 We follow the WHO definition of “young people” as individuals aged 10–24 years. Below, when presenting findings from the studies that specify participants’ ages, we use the term “adolescents” to refer to individuals aged 10–19 years and the term “youth” to refer to those aged 15–24 years, in accordance with the WHO-recommended age stratification.
