# Supplementary material for: A scoping review of psychosocial functioning in young people with personality pathology: current evidence and methodological issues
Source: Borderline Personal Disord Emot Dysregul. 2026 Jul 30;13:38. doi: 10.1186/s40479-026-00362-2 (PMC13711762; doi:10.1186/s40479-026-00362-2)
Supplement: Supplementary file 1 [file 40479_2026_362_MOESM1_ESM.docx]

Table S2. Preferred Reporting Items for Systematic reviews and Meta-Analyses extension for Scoping Reviews (PRISMA-ScR) Checklist

| **Section** | **Item** | **PRISMA-ScR checklist item** | **Information** |
| --- | --- | --- | --- |
| Title | 1 | Identify the report as a scoping review | “A scoping review of psychosocial functioning in young people with personality pathology: current evidence and methodological issues” |
| Abstract | 2 | Structured summary | **Background.** Despite evidence that personality pathology often emerges during the transition from adolescence to adulthood and leads to lasting psychosocial impairment, research on how these impairments manifest and develop in young people remains limited.  **Objectives.** This review aims to map existing literature on psychosocial functioning in individuals aged 12–25 with clinical or subclinical personality pathology, examining definitions and measures of functioning, its relationship with personality pathology, and the impact of early interventions, while identifying gaps for future research.  **Findings.** A total of 37 studies were included: 14 cross-sectional, 7 longitudinal, and 16 on early interventions. In clinical samples, overall functioning was most assessed, whereas community studies focused on specific domains. Across studies, adolescents with personality pathology showed greater psychosocial impairment than both healthy controls and other psychopathology groups, both cross-sectionally and prospectively. Improvement in global psychosocial functioning has been documented across varying interventions but there was the lack of differences between treatment approaches.  **Conclusion.** While emerging evidence highlights psychosocial dysfunction in young people with personality pathology, methodological limitations hinder comparisons across studies. Developmentally appropriate, culturally sensitive, and domain-specific assessments are needed to track psychosocial impairment alongside symptom progression in young people. |
| Introduction | 3 | Rationale | Although the association between personality disorders (PDs) and psychosocial impairment has been extensively investigated in adults, this relationship remains poorly understood during the early stages of the disorder trajectory, despite symptoms often emerging before adulthood and exerting substantial adverse effects. Little is known about how psychosocial functioning is assessed in the context of early personality pathology or which domains of functioning are most vulnerable among young people. Furthermore, the shift toward dimensional models of PD has expanded the conceptualization of the relationship between personality pathology and psychosocial functioning. To date, no review has comprehensively addressed these issues. |
|  | 4 | Objectives | The review aims to identify and document the scope of published literature examining psychosocial functioning among individuals aged 12 to 25 years with personality pathology (features of it). |
| Methods | 5 | Protocol and registration | No protocol was registered prior to conduct this review. |
|  | 6 | Eligibility criteria | Three inclusion criteria were applied: (a) young people aged 12-25 or mean age < 25); (b) indicators of (i) personality pathology ranging from symptoms (features) to PD diagnosis based in categorical and/or dimensional approaches) and (ii) psychosocial functioning or impairment); (c) examining the link of psychosocial functioning with personality pathology features or diagnosis (using correlational, longitudinal or intervention study designs). |
|  | 7 | Information sources | Scopus and PubMed; from inception to March 2026; additional search through reference lists of included papers. |
|  | 8 | Search strategy | Table 1  Table 1 provides full search string in both datanases. |
|  | 9 | Process for selecting sources of evidence | Two authors (RB & GSN) screened the titles and abstracts; two pairs of authors (RB & GSN and SP & ST) assessed the eligibility of the studies based on full texts and extracted information in the selected papers. |
|  | 10 | Data charting process | A standardized data-charting form was used. The two reviewers in the pairs independently charted the data. |
|  | 11 | Data items | Extracted information included data on study characteristics (country of origin, methodology, population and participant demographic data), construct of personality pathology (features) and its assessment method, definition (construct) of psychosocial functioning and its measure, the main results indicating (a) association (cross-sectional, longitudinal, or group differences) between personality pathology (features) and psychosocial functioning indicator(s); (b) change in psychosocial functioning (aspect) as a result of intervention. |
|  | 12 | Critical appraisal | No quality assessment performed (not applicable for scoping review). |
|  | 13 | Synthesis of results | Narrative synthesis of findings by grouping the studies by the research question. |
| Results | 14 | Selection of sources of evidence | Fig 1  PRISMA flow diagram (Fig 1); identified N = 744, included N = 37. |
|  | 15 | Characteristics of sources of evidence | Table 2  A detailed description of the characteristics of the included sources of evidence is provided, and Table 2 summarizes each study’s author, publication year, study location, research design, participant characteristics, methods, measures, and key findings. |
|  | 16 | Critical appraisal within sources of evidence | Not applicable |
|  | 17 | Results of individual sources of evidence | Table S1 (Supplement Material) presents the data and key findings based on individual sources |
|  | 18 | Synthesis of results | The results are presented narratively according to each research question, highlighting findings on: (1) the definitions and measurement of psychosocial functioning; (2) cross-sectional associations between personality pathology (or its features) and psychosocial functioning; (3) longitudinal relationships between personality pathology (or its features) and psychosocial functioning, with emphasis on bidirectional associations and trajectories of psychosocial functioning in the context of personality disorder symptoms; and (4) changes in psychosocial functioning as an outcome of interventions. |
| Discussion | 19 | Summary of evidence | This scoping review identified 37 primary studies examining the association between personality pathology (or its features) and psychosocial functioning in young people. Overall, the evidence base remains limited and is characterized by substantial heterogeneity in study design and methodology. Several key findings emerge. First, global psychosocial functioning has been the predominant outcome in clinical samples and is consistently reported as impaired, ranging from moderate to severe. Second, there is a marked asymmetry in assessment across settings: clinical studies primarily focus on global functioning, whereas community studies more often assess domain-specific outcomes, particularly social and interpersonal functioning. Third, intervention studies have largely relied on global measures of psychosocial functioning to evaluate change.  The review also highlights several methodological limitations. The predominant reliance on global measures limits understanding of domain-specific difficulties, while heterogeneity in assessment instruments reduces comparability and underscores the need for more standardized approaches across clinical and subclinical populations. In addition, the scarcity of longitudinal studies, especially in clinical samples, restricts insight into bidirectional relationships and underlying mechanisms linking personality dysfunction and psychosocial functioning in young people. The use of global functioning as the primary outcome in intervention studies may further obscure domain-specific treatment effects. Finally, evidence suggests that dimensional models of personality disorder are meaningfully associated with psychosocial functioning and should be a key focus of future research. |
|  | 20 | Limitations | Several limitations of the search strategy may have reduced the comprehensiveness of the evidence base. The review relied mainly on the term “psychosocial functioning” and broad “personality disorder” terminology, with limited use of specific subtypes, controlled vocabulary, or truncation. Additional restrictions to Title/Abstract fields, English-language, and peer-reviewed publications likely further narrowed retrieval. As a scoping review, no quality appraisal was conducted, so the robustness and consistency of findings cannot be assessed. |
|  | 21 | Conclusions | Current research on psychosocial functioning in young people with personality pathology is limited, primarily due to heterogeneous measurement approaches across clinical and community samples, which reduces comparability and complicates synthesis of findings. Evidence indicates that psychosocial impairments associated with personality disorders emerge early; however, specific indicators that predict vulnerability to impairment, particularly across distinct functional domains, remain insufficiently studied. There is also a notable gap in how psychosocial functioning is conceptualized and assessed in this population. More consistent application of DSM-5–informed functioning measures and standardized outcome sets for personality disorders (Prevolnik Rupel et al., 2021), alongside the ICF-CY framework, could improve assessment of its multidimensional nature. In addition, intervention programs for young people with personality pathology rarely prioritize domain-specific psychosocial outcomes such as school retention and social participation. No studies were identified that incorporate environmental modifications to support participation, although a promising trial has recently been initiated (Chanen et al., 2020). This is particularly concerning during adolescence, a critical developmental period, as insufficient attention to the multidimensional nature of psychosocial functioning may hinder the prevention of long-term disability and increase societal and economic costs. |
| Funding | 22 | Sources of funding | No funding was received. |
