# Supplementary material for: A scoping review of psychosocial functioning in young people with personality pathology: current evidence and methodological issues
Source: Borderline Personal Disord Emot Dysregul. 2026 Jul 30;13:38. doi: 10.1186/s40479-026-00362-2 (PMC13711762; doi:10.1186/s40479-026-00362-2)
Supplement: Supplementary file 2 [file 40479_2026_362_MOESM2_ESM.docx]

Table S2. Study results

| **Author (year)**  **Country**  **Study design**  **Population** | **Personality pathology (feature) construct** | **Psychosocial functioning domain** | **Results** |
| --- | --- | --- | --- |
| Amminger et al. (2013)  Austria  Intervention study  Clinical | BPD diagnosis | Global level of functioning (GAF) | Improvement in GAF (from 55.7 to 75.6; Cohen d = 1.59) among patients with both BPD and ultra-high risk for psychosis, assigned to receive either 0.5 g of concentrated marine fish oil vs placebo. |
| Barkauskienė et al. (2021)  Lithuania  Cross-sectional  Community | Self-reported BPD symptom level | Academic functioning  Social functioning  Other (life satisfaction) | The subgroup of high borderline personality (BP) symptoms profile (19.7% of the sample) had the highest level of social problems, a smaller number of close friends, lower academic motivation and achievement, and the lowest life satisfaction as compared to those with low and/or moderate levels of BP symptoms profiles.  BP symptoms correlated with social problems (r=.72), number of close friends (r= -.19), achievement (r=-.12), academic motivation (r= -.22), and life satisfaction (r=-55).  Controlling for gender and psychopathology, BP symptoms added 1.1% of variance to social problems, 1.2% to lower achievement, and 2.8% to life satisfaction. |
| Barkauskienė et al. (2024)  Lithuania  Cross-sectional  Clinical and community | Self-reported BP features; Self-reported level of personality functioning (Criterion A) and maladaptive traits (Criterion B) | Social functioning | Clinical sample: large correlation of social problems with BP features, Criterion A, and Criterion B (r = .66, .71, and .74, respectively); Criterion A explains 7.8% of unique variance in social problems (controlling for age, gender, and Criterion B)  Community sample: large correlations of social problems with BP features, Criterion A, and Criterion B (r = .59, .66, and .61, respectively); Criterion A explains 7.8% of unique variance in social problems (controlling for age, gender, and Criterion B) |
| Barkauskienė et al. (2025)  Lithuania  Follow-up  Community | Self-reported level of personality functioning (Criterion A) and maladaptive traits (Criterion B) | Academic functioning  Social functioning  Other (life satisfaction) | Baseline correlations between both Criterion A and Criterion B and functioning measures varied from small (academic motivation, social rebuff) to large (social difficulties, life satisfaction)  Longitudinal associations between baseline (T1) Criteria A and B remained statistically significant and of comparable strength with psychosocial functioning indicators at one-year follow-up (T2)  Controlling for gender, psychopathology, and respective T1 functioning assessment, Criterion A had a unique association with T2 social difficulties, social rebuff, and life satisfaction; Criterion B psychoticism domain added to social rebuff only. A total score of Criterion B uniquely explained social difficulties. Neither Criterion A nor Criterion B had a unique association with T2 academic motivation. |
| Baumer et al. (2022)  Switzerland,  Germany  Intervention study  Clinical | BPD symptoms count (clinician rated) | Global level of functioning | GAF change in total sample from M=48.8 (SD=7.2) to M=64.4 (SD=1.3), with no difference between DBT-A & AIT; expectancy on outcome correlated with a higher global level of functioning at follow-up. |
| Beck et al. (2020)  Denmark  Intervention study  Clinical | BPD criteria count (clinician interview-based) | Global level of functioning | GAF at baseline was 35.2 in the TAU condition and 35.7 in the MBT-G group, corresponding to 'major impairment in functioning'. At the end of treatment, functioning moved to the level of 'moderate impairment in functioning" (TAU-46.7; MBT-G-46.1); no difference between conditions. |
| Beeney et al. (2021)  United States  Follow-up  Community | Self-reported BPD symptoms | Global level of functioning  General impairment in academic domain | Global functioning at age 14-15 was a significant predictor of BPD symptoms at age 16-18 (along with other 18 variables, spanning 13 constructs), with the effects of functioning being evident from age periods of 8-10 and 11-13 as well. No effect of earlier functioning on BPD symptoms at age 14-15 was detected. No significant effect of general impairment in academic domain (as rated by teachers) was identified. |
| Biskin et al. (2021)  Canada  Follow-up  Community | Self-reported NSSI and BPD symptom levels | General functioning level | Those who continued engagement in NSSI reported the highest level of general functioning impairment and BPD symptoms, compared to those who never engaged in or stopped NSSI. |
| Calvo et al. (2025)  Spain  Cross-sectional  Clinical and community | BPD diagnosis and number of symptoms (clinician-rated)  Self-reported BP features | General functioning and subdomains (autonomy, occupational, cognitive, financial issues, interpersonal, and leisure) | Young people with BPD as compared to healthy controls obtained higher scores in the total FAST score and all subdomains. Adolescents with BPD had less difficulties than adults in autonomy, occupational, and interpersonal functioning; did not differ on leisure, financial, cognitive issues; severity of BPD (measured with BPFSC-11) was linked to global FAST score, autonomy, cognitive, financial, interpersonal domains in adolescents; none statistically significant association was found in young adults. |
| Cavelti et al. (2024a)  Switzerland, Germany  Intervention study  Clinical | Number of BPD criteria (clinician-rated) | Global level of functioning | Improvement in functioning irrespective of group (received CDP only, accepted DBT-A+, and declined DBT-A-); gains in functioning continuously increased for CDP group only. No clinical differences in functioning at follow-up (two and three years after baseline) between those who accepted (DBT-A+) or declined (DBT-A-). |
| Cavelti et al. (2024b)  Switzerland, Germany  Intervention study  Clinical | Number of BPD criteria (clinician-rated) | Global level of functioning | In adolescents, who received any psychiatric or /psychotherapeutic treatment, significant improvement in GAF from baseline (T0) to T1 (one-year follow-up) and between T1 and T2 (two years after baseline); moderated by group - greater in outpatients that in combined group of in- and outpatients; outpatients had greater increase T0-T1 but comparable T1-T2 |
| Cavelti et al. (2025)  Switzerland, Germany  Cross-sectional  Clinical | Level of personality functioning (clinician-rated) | Global level of functioning | In females, personality dysfunction (Criterion A) is associated with functioning stronger than in males (β = −3.44, 95% CI = [−6.84, −0.04], p =0.047). |
| Chanen et al. (2007)  Australia  Cross-sectional  Clinical | BPD and other PD diagnoses | Global level of functioning  Academic functioning  Social functioning  Daily functioning | BPD patients, compared to psychiatric controls, had more impaired peer and family relationships, daily and global functioning; the same pattern was detected for patients with other PDs.  BPD and other PD did not differ on global functioning (M=60.04, SD=10.80 vs M=59.50, SD=22.06). All three groups did not differ on school attendance; BPD vs other PDs had more days out or role due to truancy.  BPD uniquely predicted daily functioning (after controlling for Axis I and other PDs) and together with other PDs predicted global functioning and peer relationships. |
| Chanen et al. (2008) | BPD symptoms count | Global functioning | Cognitive Analytic Therapy (CAT) vs good clinical care (GCC): both treatment groups demonstrated improvements in global functioning as assessed by SOFAS from baseline (CAT: M = 60.27, SD = 8.40; GCC: M = 61.16, SD = 10.53) to 24-months follow-up (CAT: M = 71.74, SD = 11.56; GCC: M = 75.30.16, SD = 12.21), with the expected rate of improvement being moderately fasted for GCC; no noticeable difference between groups in global functioning was detected at 2-years follow-up. |
| Chanen et al. (2022)  Australia  Intervention study  Clinical | BPD diagnosis | Social functioning | Improvement over a 12-month period, with a 19.3 to 23.8 % drop in social functioning impairment scores. Neither of the treatment conditions (service model (HYPE vs YMHS) or intervention (CAT vs befriending) was superior to the change in functioning. Global level of functioning at the baseline (based on SOFAS) was 57.9 (SD = 8.0); NEET status – 43% |
| Chanen et al. (2024)  Australia  Cross-sectional | BPD diagnosis | Global level of functioning | The BPD+FEP group had the lowest global functioning (M=47.9, SD=12.7)  compared to clinical controls (M=68.5, SD=10.5) and FEP (M=58.7, SD=12.5), but did not differ from BPD group (M=54.2, SD=8.8) |
| Ghinea et al. (2021)  Germany  Cross-sectional  Clinical | BPD symptoms count (clinician rated) | Global level of functioning | Global functioning scores: M = 47.89 (SD = 10.94) in total sample; M = 50.93 (SD = 12.13) in NSSI-CM, and M = 46.64 (SD=10.18) in NSSI+CM; p<.001.  BPD criteria number: M = 4.08 (SD = 2.17) in NSSI+CM vs M = 2.94 (SD = 2.05) in NSSI-CM. |
| Gleeson et al. (2012) | BPD symptoms count | Global functioning | In patients, aged 15-25 and meeting both BPD and first-episode psychosis, for HYPE+SFET group improvement in global functioning as assessed by SOFAS has been achieved at the end of treatment (from M = 52.9, SD = 12.4 (baseline) to M = 67.0, SD = 8.3) and 6-months follow-up (M = 69.3, SD = 4.3). |
| Gleeson et al. (2021) | BPD severity | Global functioning | Mean global score improved at follow-up, (from baseline M = 17.4, SD = 7.5 to M = 11.0, SD = 2.9) with 60% reliably improved score and 0% reliably worse score; the change was statistically insignificant, but of large magnitude (Cohen's d =−1.047, 95% confidence intervals−2.37 to 0.28). |
| Hessels et al. (2024)  Netherlands  Cross-sectional  Clinical | Self-reported BPD symptoms count; self-reported level of personality functioning (Criterion A) and maladaptive traits (Criterion B) | Age adequate functioning | All personality pathology constructs had medium correlations with general age-appropriate functioning.  All personality pathology constructs explained 41.9% of variance in psychosocial functioning; with Criterion A (self and interpersonal) & Criterion B (negative affectivity, disinhibition, and detachment traits) being significant predictors; Criterion A and Criterion B explained more variance in functioning (34.3 and 39.1% respectively) as compared to BPD symptoms count (17.5%). |
| Jorgensen et al. (2021)  Denmark  Intervention study  Clinical | BPD criteria count (clinician-rated) | Global level of functioning | GAF score ranged from 46.7 (at the end of treatment) to 46.5 (at 3 months follow-up) and 50.4 (at 12 months follow-up) in MBT-G condition and did not differ significantly from TAU condition (46.1 (at the end of treatment) to 46.0 (at 3 months follow-up) and 50.1 (at 12 months follow-up)).  The level of global functioning remained in the category of moderate degree of interference in functioning in both conditions at both follow-ups. |
| Jorgensen et al. (2024)  Denmark  Follow-up  Clinical | PD diagnosis | General functioning level | In the five-year longitudinal, naturalistic follow-up study of adolescents who received MBT-G intervention, the level of global functioning (assessed with WSAS; M = 13.39, SD = 8.9) corresponded to the level of moderate functional impairment (based on normative data in a Danish sample); NEET status for 36% of the sample. |
| Kaess et al. (2012)  Germany  Cross-sectional  Clinical | BPD diagnosis | Global level of functioning | The BPD group had lower global functioning compared to clinical controls (M = 38.71, SD = 6.05 vs M = 48.39, SD = 13.28).  Psychosocial functioning was predicted by socioeconomic status and comorbid disorders, as well as the unique influence of BPD diagnosis itself. |
| Kramer et al. (2017)  United States  Cross-sectional  Clinical | BPD diagnosis | Global level of functioning  Interpersonal functioning  Occupational (vocational) functioning | In adolescents with BPD, global functioning is lower by ~40 points compared to healthy controls, with more impairments in interpersonal functioning (relations with parents, more dating, more time alone) and occupational/vocational (not good work, history of schooling, more expulsions from school, less community participation) domains. No differences in relations with friends. |
| Mehlum et al. (2014)  Norway  Intervention study  Clinical | Number of BPD criteria | Global level of functioning | Level of functioning at baseline: M = 56.1, SD = 8.3 (total sample); M = 55.3, SD = 8.0 (DBT-A group), and M = 57.9, SD = 10.1 (EUC group). |
| Mehlum et al. (2016)  Norway  Intervention study  Clinical | Number of BPD criteria | Global level of functioning | At 1-year follow up, no further improvement in DBT-A participants (from M = 65.88, SD = 9.52 at the end of treatment (19-week) to M = 65.65, SD = 11.81 at 71-week); inter-group outcomes in global functioning apparent at the end of treatment (19-week) were no longer observed. |
| Mehlum et al. (2019)  Norway  Intervention study  Clinical | Number of BPD criteria | Global level of functioning | At 3.1-year follow-up, both groups (DBT-A and enhanced usual care (EUC)) remained on average at the same levels as at the 1.6 years follow-up (DBT-A: from M = 65.65, SD = 11.8 to M = 64.97, SD = 11.75; EUC: from M = 64.22, SD = 14.13 to M = 66.12, SD = 11.19); no significant differences between treatment groups. |
| Palermo et al. (2026) | BPD criteria count | Global level of functioning | Bbaseline psychosocial functioning did not predict diagnostic remission or overall changes in personality functioning, but higher baseline functioning was associated with less improvement in self-direction (β = 0.008, p = .033). |
| Schmeck et al. (2023)  Switzerland, Germany  Intervention study  Clinical | BPD symptoms count (clinician-rated) | Global level of functioning  General functioning level | Both treatment approaches (AIT and DBT-A) improved adolescents’ psychosocial functioning in a comparable way and with very large effect sizes (AIT: dz = 1.82; DBT-A: dz = 1.73); self-reported general functioning also improved, as well as parent-rated, with lower effect sizes; recovery (C-GAS > 60) was achieved by 69.2 (AIT) and 53.8 (DBT-A) patients. |
| Sharp et al. (2022)  Lithuania  Cross-sectional  Community and clinical | Self-reported BPD symptom level | Academic functioning  Social functioning  Other (life satisfaction) | In the full sample (community and clinical groups combined), borderline features correlated with peer problems (r = .42, p<.001), life satisfaction (r = -.60, p<.001), and academic motivation (r = -.25, p<.001; peer problems and life satisfaction remained statistically significant unique predictors of borderline features, (b= .08 and b = .06, respectively), controlling for age, gender, general psychopathology, maladaptive identity, and academic motivation. |
| Thompson et al. (2019)  Australia  Cross-sectional  Clinical | Number of BPD criteria | Global level of functioning  Days out of role | Groups with 0 and 1-4 BPD features had statistically significant differences in overall/global functioning (M=69.67, SD=13.0 vs M=63.3, SD=11.65; t = 3.57, p<.0001), but not in the number of days out of role in the past 6 months (N=31.44, SD=40.32 vs N=33.20, SD=38.72) |
| Thompson et al. (2020)  Australia  Cross-sectional  Clinical | Number of BPD criteria (clinician-rated) | Global level of functioning | Global functioning level M = 61.77 (SD = 12.14) in a sample of clinical youth with a subthreshold number of BPD criteria (M=3.49, SD=2.58); moderate correlation (r = .43) of BPD and global functioning.  BPD uniquely explains 11.7% of the global level of functioning along with sex (female), current depression, and service utilization, (each adding up to 3%). |
| Wertz et al. (2020)  Great Britain  Follow-up  Community | BP symptoms (interview with mothers-based) | Social functioning  Academic functioning  Other (NEET status, crime records, life satisfaction) | Those with higher levels of BP symptoms at age 12 had worse functioning outcomes at age 18 (poorer educational and societal outcomes (NEET status), lower life satisfaction, more social isolation, and were more likely to have broken the law). BP symptoms uniquely predicted low educational qualifications (IRR=1.18) and official crime records (IRR=1.19) (controlling for sex and initial levels of psychopathology at age 12). |
| Winograd, Cohen & Chen (2008)  United States  Follow-up  Community | Self-reported level of borderline symptoms | Social (interpersonal functioning)  Occupational functioning  Other (life satisfaction) | Early borderline symptoms predicted lower average role function (β = -.107, p<.001); lower social support (β = -.162, p<.001), and life satisfaction (β = -.181, p<.001), but not relationship quality (β = -.059, p=.075).  Men reported notably lower relationship quality, lower social support; men and those older at baseline - lower levels of life satisfaction.  During 10 years follow-up, role function increased (more rapidly up to 23.4 y.o) but those with diagnostic level of early BPD (2 SD above mean) had persistently lower role function with average difference of Cohen’s d = .22; relationship quality (with closest person) was stable; social support declined over all period with Cohen’s d=.32 difference between mean and diagnostic level symptoms; life satisfaction declined up to mid-20s and the difference between mean level and diagnostic level was Cohen’s d = .36).  Past role function tends to predict subsequent, and ~30% of the association between role function and BP symptoms is attributable to this covariate. |
| Wright et al.  (2016)  United States  Follow-up  Community | Self-reported BPD symptoms | Global level of functioning  Academic functioning  Occupational functioning  Social (interpersonal) functioning  Other (sexual behavior) | Individual differences in initial BPD symptom levels (intercept factors) correlated with all functioning domains (Mdn r = .50; range = .21–.61). Changes in BPD (growth factors) were associated with increases in psychosocial dysfunction (Mdnr = .44, range = .29–.74).  The unique links in the trajectories of BPD symptoms and functioning were for self-reported social skills, sexual activity, and self-perception (self-worth, self-confidence, and peer-victimization) (controlling for depression and conduct disorder). Across waves, no change in self-perception, but GAF, social skills, and problems in achievement increased, and involvement in extracurricular activities decreased. |
| Wyssen et al. (2024)  Germany  Cross-sectional  Clinical | BPD diagnosis; Impaired level of personality functioning (Criterion A) (clinician-rated) | Global level of functioning | Criterion A score and BPD correlated with overall functioning with medium correlations (r=-.47 vs r=-.38; p<.001)  Exceeding the diagnostic threshold on at least one measure (Criterion A, BPD or Criterion A+BPD) had more psychosocial impairment than non-PD groups.  The BPD+Criterion A group had the highest global functioning impairment, followed by Criterion A and the BPD groups. Among diagnostic groups, only BPD and BPD+Criterion A differed in functioning levels. |
| Zanarini et al. (2008)  United States  Intervention study  Clinical | BPD diagnosis | Social functioning  Vocational functioning | Immediate education about BPD did not improve psychosocial functioning in both social and vocational domains |

*Note:* DBT-A = Dialectic Behavior Therapy for Adolescents; AIT = Adolescent identity Treatment; MBT-G = Mentalization Based Therapy in groups; TAU = Treatment as usual; CDP = Cutting Down Program; HYPE = Helping Young People Early; YMHS = Youth Mental Health Service; CAT - Cognitive Analytic Therapy; FEP = First episode of psychosis; NSSI = Non-suicidal self-injury; CM = Childhood maltreatment; WSAS = The Work and Social Adjustment Scale; NEET = not in education, employment, or training; IRR = incidence rate ratios
